# Predicting the outcome of plantar heel pain in adults: a systematic review of prognostic factors

**DOI:** 10.1186/s13047-023-00626-y

**Published:** 2023-05-12

**Authors:** Halime Gulle, Dylan Morrissey, Xiang Li Tan, Matthew Cotchett, Stuart Charles Miller, Aleksandra Birn Jeffrey, Trevor Prior

**Affiliations:** 1 Sports and Exercise Medicine, William Harvey Research Institute, Bart’s and the London School of Medicine and Dentistry, Queen Mary University of London, Mile End Hospital, Bancroft Road, London, E1 4DG UK; 2 Department of Rheumatology, Medicine, Ashford and St Peter’s Hospital, Guildford St, Lyne, KT16 0PZ Chertsey UK; 3 Department of Physiotherapy, Podiatry, Prosthetics and Orthotics, La Trobe University, Melbourne, Australia; 4 School of Engineering and Materials Science, Institute of Bioengineering, Queen Mary University London, Mile End, Bancroft Road, London, E1 4DG UK; 5 Consultant Podiatric Surgeon Homerton University Hospital, Homerton Row, London, E9 6SR UK

**Keywords:** Prognosis, Outcome predictors, Plantar heel pain

## Abstract

**Background:**

Plantar Heel Pain (PHP) is a common disorder with many treatment pathways and is not self-limiting, hence prognostic information concerning recovery or recalcitrance is needed to guide practice. In this systematic review, we investigate which prognostic factors are associated with favourable or unfavourable PHP outcomes.

**Methods:**

MEDLINE, Web of Science, EMBASE, Scopus and PubMed electronic bibliographic databases were searched for studies evaluating baseline patient characteristics associated with outcomes in prospective longitudinal cohorts or after specific interventions. Cohort, clinical prediction rule derivation and single arms of randomised controlled trials were included. Risk of bias was evaluated with method-specific tools and evidence certainty with GRADE.

**Results:**

The review included five studies which evaluated 98 variables in 811 participants. Prognostic factors could be categorised as demographics, pain, physical and activity-related. Three factors including sex and bilateral symptoms (HR: 0.49[0.30–0.80], 0.33[0.15–0.72], respectively) were associated with a poor outcome in a single cohort study. The remaining four studies reported twenty factors associated with a favourable outcome following shockwave therapy, anti-pronation taping and orthoses. Heel spur (AUC = 0.88[0.82–0.93]), ankle plantar-flexor strength (Likelihood ratio (LR): 2.17[1.20–3.95]) and response to taping (LR = 2.17[1.19–3.90]) were the strongest factors predicting medium-term improvement. Overall, the study quality was low. A gap map analysis revealed an absence of research that included psychosocial factors.

**Conclusions:**

A limited number of biomedical factors predict favourable or unfavourable PHP outcomes. High quality, adequately powered, prospective studies are required to better understand PHP recovery and should evaluate the prognostic value of a wide range of variables, including psychosocial factors.

**Supplementary Information:**

The online version contains supplementary material available at 10.1186/s13047-023-00626-y.

## Background

Plantar heel pain (PHP) is one of the most troublesome and common foot conditions, with an estimated prevalence between 4 and 10% in the general population [1–4]. PHP is characterised by pain in the inferior-medial aspect of the rear-foot that is typically worse upon weight-bearing activities such as walking or standing or on weight bearing after periods of rest, and palpation of the medial tubercle of the calcaneus [4]. Consequently, PHP can have a negative impact on health-related quality of life, including limiting activities of daily living and contributing to social isolation [5].

Multiple treatment options are available for PHP. A recent comprehensive systematic review recommended stretching, taping and patient education in first-line management, with interventions such as shock wave therapy, foot orthoses and injections for those who fail to improve [6]. However, PHP can still remain resistant to treatment, and although some studies have reported high levels of spontaneous recovery within one year [7, 8], there is evidence of up to 50% recalcitrance at 10 years [9]. Multiple treatment options with unsatisfactory results may arise from the lack of tailoring management strategies due to limited understanding of the biopsychosocial factors that affect PHP prognosis. Prognostic factors are variables at baseline which are associated with a subsequent outcome of pain, function and disability, and can be evaluated with specific research designs such as prospective cohort studies, analysis of single arms in randomised controlled trials (RCTs) and clinical prediction rule derivation studies. To inform clinical care and delivery and to better understand the likely course of an individual’s condition, there is a need to identify and evaluate prognostic factors.

In other musculoskeletal conditions, such as patellofemoral pain, prognostic factors such as a disease duration of longer than 2 months, lower scores on an anterior knee pain scale and higher activity-related pain may predict those who have a poor 12-month prognosis [10]. A recent systematic review of prognostic factors in tendinopathy showed that limited evidence exists linking psychological variables and tendinopathy, and suggested that using validated screening tools for the presence of psychological variables should be a part of their holistic management [11]. While there are multiple systematic reviews and clinical practice guidelines that have evaluated the effectiveness of interventions for PHP there is no review of prognostic factors for PHP.

We aimed to inform clinical care for PHP by 1) determining which baseline patient characteristics are associated with outcomes in observational cohorts or after specific interventions, and 2) analysing the quality of the available research and the gaps within it (i.e. identify biomedical, physical and psychosocial variables that have yet to be investigated). This second aim will influence best practise and help researchers who want to work in the field of PHP prognosis and direct their efforts more effectively by guiding future work to improve our understanding of outcomes for this troublesome, common and recalcitrant condition.

## Methods

The Preferred Reporting Items for Systematic reviews and Meta-Analyses (PRISMA) guidelines were followed [12], and a published guideline for design and reporting of systematic reviews of prognostic factor [13]. The review protocol was registered on PROSPERO (CRD42020205005).

### Search strategy

Electronic databases (Ovid MEDLINE, Scopus, Embase, Pubmed and Web of Science) were searched from inception to June 2020. Key search terms used in the selection process relating to PHP were [plantar heel pain OR plantar fasci* OR heel pain syndrome], which were adapted from previous studies with similar search strategies [6, 14]. Keywords of [success*, factor*, predict*, charact*, prognos*] were used in combination with the keywords related to PHP, in order to capture primary prognostic research [15]. The complete search strategy is reported in the electronic supplementary material.

### Eligibility criteria

Studies investigating baseline characteristics with follow-up of patient-reported outcomes relating to indicators of recovery (e.g. pain and/or function) after at least one week were included. Studies were also required to clearly define recovery and provide an effect size for the prognostic estimate. Prospective cohort studies, single arm clinical trials reporting prognostic factors and studies [16] developing clinical prediction rules were included. The inclusion and exclusion criteria are presented in Table 1.

**Table 1:** Inclusion criteria for eligible studies

Inclusion criteria
Design:
• Prospective cohort study; single arm clinical trials or clinical prediction rule derivation study;
Participants
• Inferior heel pain, that is pronounced with weight bearing or upon weight bearing after periods of rest and pain in palpation of the medial tubercle of the calcaneus for more than 1 month
Main outcome and outcome measures
• Recovery of plantar heel pain by measuring pain and function (i.e. VAS, FFI, GROC, PSFS)
Measures of effect size:
• At least one possible effect size measure e.g. odds ratios, risk ratios, hazard ratios, positive likelihood ratio, and area under curve
Language:
• No restrictions, with translators readily available

Key: *VAS* Visual analog scale, *FFI* Foot function index, *GROC* Global rating outcome scale, *PSFS* Patient specific functional scale

No publication date limits, or language restrictions were set RCTs that were not considered single arm prognostic research studies were excluded, as were retrospective studies due to the low level of evidence [16].

### Types of participants

Studies which investigated adult participants over 18 years of age with a clinical diagnosis of PHP were included. To be consistent with previously published criteria [17], we included participants with a diagnosis of PHP of greater than one month duration that is worse on weight bearing, or on weight bearing after periods of rest, and palpation of the medial tubercle of the calcaneus. Studies including participants without a clear diagnosis of PHP, and/or describing pain in other body areas, including other foot pathologies were excluded (Table 1).

### Review process

Identified studies were imported into Endnote X6 (Thomson Reuters, Carlsbad, California, USA) where duplicates were removed, before uploading to Rayyan QCRI (Computing Research institute, QATAR) for title and abstract screening. Two reviewers independently assessed study titles and abstracts, screened full-texts, verified eligible papers, and completed the quality assessments. A third reviewer (HG and XL) was available for difficult decisions and to resolve discrepancies.

### Data extraction and data synthesis

Data were extracted from studies on September 2020 according to the CHecklist for critical Appraisal and data extraction for systematic Reviews of prediction Modelling Studies (CHARMS) [18]. As studies had different durations, follow-up lengths were categorized as short term (range 0–12 weeks), intermediate (13–26 weeks) and long term (1 year, ≥ 52 weeks), and long term (≥ 52 weeks) [6].

All results, including non-significant prognostic factors, were extracted from each study. Any prognostic factor investigated by multiple studies for different time periods, effect measures and scores (e.g., Hazard ratio (HR), + Likelihood ratio (LR +), Area Under Curve (AUC) and *P* value) and level of evidence, were tabulated and presented graphically as a gap map in table 4. It was also included psychological and social contextual (cultural) factors potentially relevant when analysing a patient’s presenting problems [19].

According to recommendations by Riley et. al (2019), meta-analyses were not performed due to the diverse range of effect sizes, study methodologies, and adjustments for covariates.

### Quality assessment

Study quality was evaluated using the Quality Assessment of Diagnostic Clinical Prediction Rules (QUADCPR) which consists of 23 items divided into four sections, with each item scoring yes (score = 2), no (score = 0) or unclear (score = 1), which makes 54 of total score. The first section includes a checklist of items related to the sample and participants; the second section focuses on the reporting of outcome measures; the third section relates to the quality of tests and measures; and the final section focuses on the quality of reporting related to statistics [20].

The Epidemiological Appraisal Instrument (EAI) includes 43 items which are scored as yes (score = 2), partial (score = 1), no (score = 0) or unable to determine (score = 0). Questions 10, 22, 23, 24 were removed as they are not applicable to intervention studies. The EAI has proven to be a valid and reliable evaluation method that can be used in different applications, such as systematic evaluations and meta-analyses [21].

### Risk of bias assessment

The Quality In Prognosis Studies (QUIPS II) was used to tool has been found to be useful and reliable for systematic reviewers, study authors, and readers to guide comprehensive assessment of bias in studies of prognostic factors [22]. It includes 24 items across 6 domains including study participation, study attrition, prognostic factor measurement, outcome measurement, study confounding, statistical analysis and reporting. The overall assessment of the six risk of bias domains judgments were scored as yes, partially, unsure or no [23]. Each of the six domains were rated independently by two reviewers (HG and XL).

## Evaluation of the quality of evidence using GRADE

Evidence levels were established based on the modified Grading of Recommendations Assessment, Development and Evaluation (GRADE) framework [24]. The phase of investigation was considered as a starting point. As all included articles were categorised as phase 1 explanatory studies, they were judged as moderate level of evidence at the beginning according to recommendations [24]. Afterwards, the evidence level was downgraded based on the following descriptions:

1. Study limitations: a) serious limitations when most evidence is from studies with moderate or unclear risk of bias for most bias domains; b) very serious limitations when most evidence is from studies with high risk of bias according to QUIPS II.
2. Inconsistency: variations in effect estimates across studies or providing different results for the same variables.
3. Imprecision (within-study imprecision): a) sample size justification is not provided and there are less than 10 outcome events for each prognostic variable b) points of effect on either side of the line of no effect, and confidence intervals showing minimal overlap.
4. Publication bias: all studies are downgraded in this item due to their study phase [24, 25]. Evidence level were upgraded based on the following descriptions:
  a Moderate or large effect: a moderate or large similar effect is reported in the study.

**Fig. 1:**
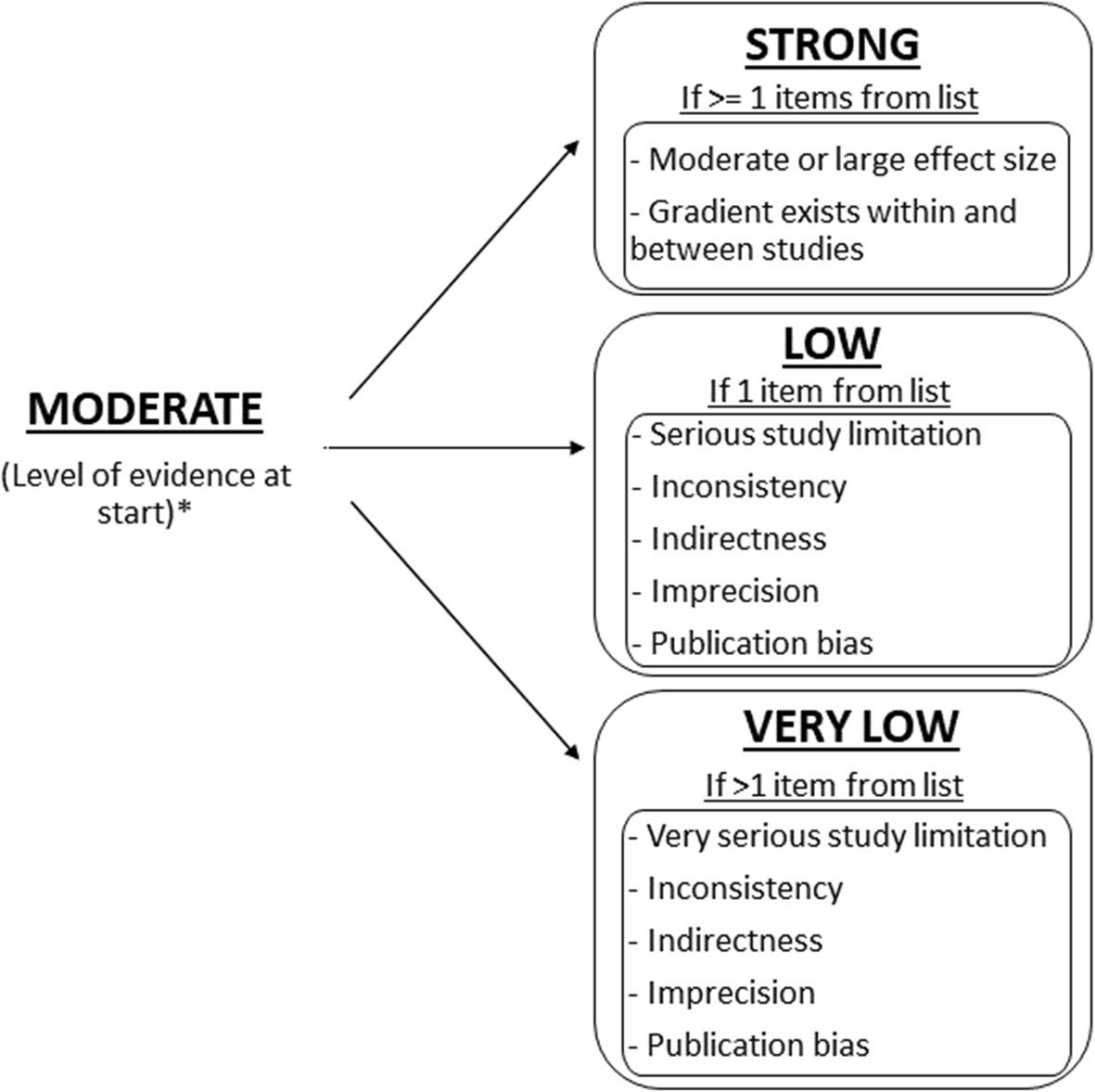
Guide for adjustments to the quality of evidence for prognosis; This diagram is adapted from Huguet et. al (2013). * In this review, moderate level of evidence is the starting point for outcome prediction research or explanatory research aimed to identify associations between potential prognostic factors and the outcome (Huguet et. al., 2013)

Figure 1 presents a guide for adjustments to the quality of evidence using criteria outlined by Huguet et al., (2013) [24]. A range of effect size measures were determined based on previous literature, including Hazard Ratios (HRs), Likelihood Ratios (LRs) and Area Under the Curve (AUC) Small, medium, and large Hazard Ratios (HRs) for a standard deviation increase in the predictor were classified as 1.14, 1.47, and 1.9, respectively [26]. Likelihood ratios (LRs) LR + 5–10 represents moderate probability; LR + 2–5 generate small but important probability; LR + 1–2 generate small but rarely important probability [27]. Regarding the AUC, a ROC = 0.5, suggests no discrimination; 0.7 < ROC < 0.8 is considered acceptable; 0.8 < ROC < 0.9 is considered excellent and if the ROC > 0.9 it is considered outstanding [28].

## Results

### Search results and critical appraisal of methods

**Fig. 2:**
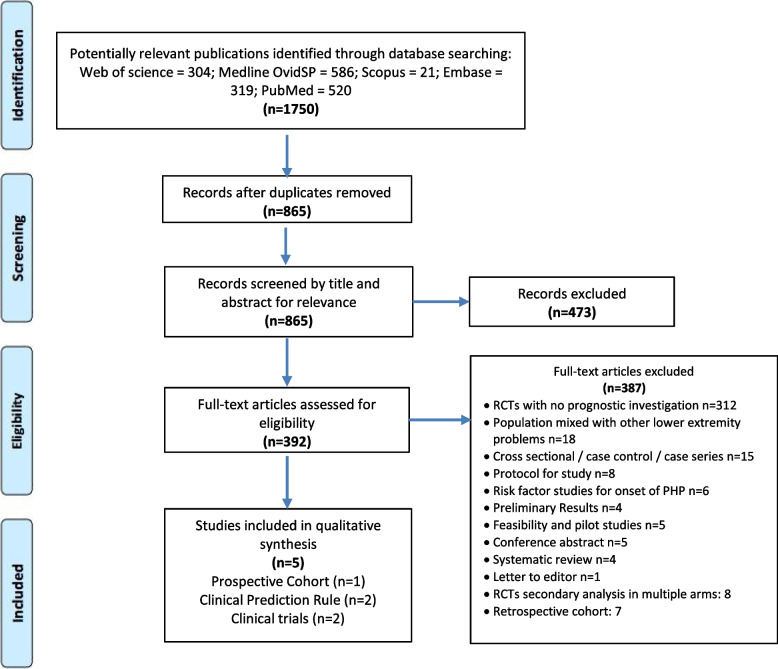
PRISMA flow diagram; Key: n = number, RCTs = Randomized controlled trials

After search strategy, we included 5 studies (Fig. 2) investigating 811 participants with PHP in total (range = 74 to 278). The results of the quality assessment, a summary of the included 225 studies and outcome predictors are presented in Table 2, Table 3, and Table 4, respectively.

**Table 2:**
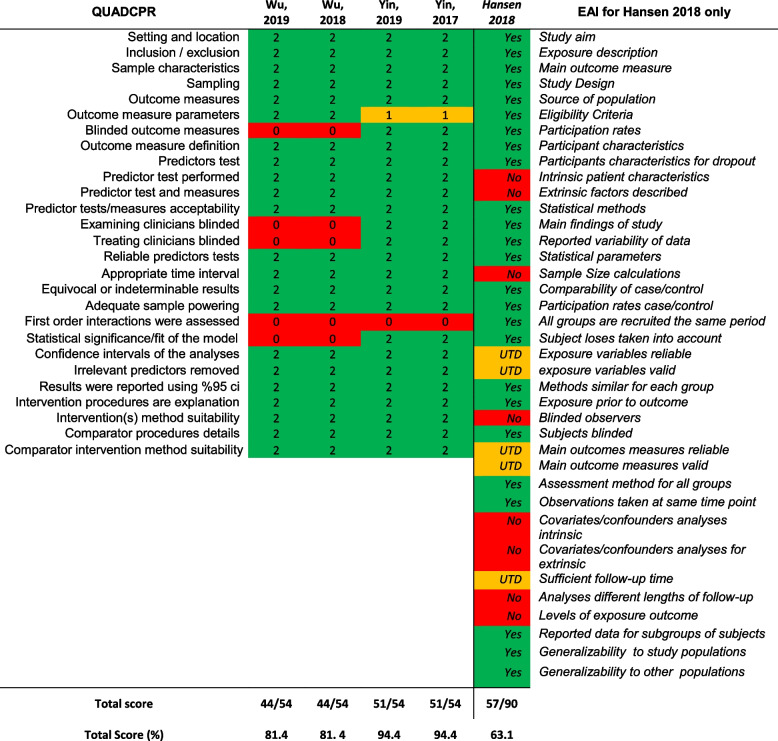
Quality assessment of studies using QUADCPR and EAI

Key = *UTD* Unable to Detect, *QUADCPR* Modified Quality Assessment of Diagnostic Clinical Prediction Rule, *EAI* The Epidemiological Appraisal Instrument; 2 = yes; 1 = unclear; 0 = no. Inter-rater agreement between the quality assessors was 82% across all 5 papers

Inter-rater agreement between the quality assessors was 92% across all 13 papers

^*^Modified in accordance with the TRIPOD statement23 recommendation for a minimum of 10 participants in the limiting sample size (ie, those who experienced the least frequent outcome) for each potential predictor variable included in the analysis

**Table 3:** Characteristics of 5 included studies

**Study**		**Participants**		**Treatment**		**Outcomes to be predicted**		**Analysis**	
**Study and design**	**Ss and events**	**Demographics (age, BMI, gender f:m)**	**Prescribed Treatments**	**Permitted Treatment**	**Positive Outcome criteria**	**Follow-up length**	**Modelling method**	**Factors (n)**	**Prognostic factors identified**
Hansen, 2018 Cohort study	174 80	Age: 26—88 years, BMI: 17.8—43.3 kg/m2 Sex: 91 (52%): 83 (48%)	93% US-guided injections	Various physiotherapy modalities	Scored > 0 on the NRS in either rest, during walking, during running, or on pressure	5 to 15 years	Multiple Cox regression analysis	9	1. Gender 2. Bilateral heel pain
Wu, 2019, Clinical Prediction Rule	75 49	Age: 48.4 ± 14.5 years, BMI: 23.8 ± 3.7 kg/m2 Sex: 57 (77%):17 (23%)	Customized foot orthosis	Not allowed	(1) Reducing the pain intensity > 2 points or 50%, (2) Decreasing the FFI score > 7 points or PSFS score > 50% (3) Improving GROC scale of + 4	6 months	Multiple logistic regression	63	1. Change in pain after taping, 2. Range of ankle PF > 54°, 3. Unbalanced strength of ankle PF, 4. Range of hip IR < 39°, 5. Range of hip ER > 45°
Wu, 2018, Clinical Prediction Rule	74 28	Age: 48.4 ± 14.5 years, BMI: 23.8 ± 3.7 kg/m2 Sex: 58 (77%): 17 (23%)	Anti-pronation taping	Not Reported	(1) Reducing the pain intensity > 2 points or 50%, (2) Decreasing the FFI > 7 points or PSFS score > 50% (3) Improving GROC scale of + 4	1 week	Multiple logistic regression	79	1. FFI score less than 33.3, 2. Unbalanced hip adduction angle, 3. Unbalanced ankle PF and hip abductors, 4. Unbalanced on ankle invertors, 5. > 2 painful sites in lower extremity regions
Yin, 2017, Clinical Trial	278 186	Age: 55 ± 13.3 years, BMI:107(38.5%) < 26 kg/m2, 147 (52.9%) 26–30 kg/m2 24 (8.6%) > 30 kg/m2 Sex: 136(49%):142(51%)	ESWT	Not Reported	Reducing the pain intensity > 2 points or 50%,	3 months	Multiple stepwise logistic regression	10	1. VAS, 2. Heel spurs 3. Oedema
Yin, 2019, Clinical Trial	210 140	Age: 54.1 ± 13.6 years, BMI: 76(36.2%) < 26 kg/m2, 112 (53.3%) 26–30 kg/m2 22 (10.5%) > 30 kg/m2, Sex: 98 (47%):112(53%)	ESWT	Not Reported	Reducing the pain intensity > 2 points or 60%	6 months	Artificial neural networks	10	1. VAS, 2. Heel spurs 3. Duration of symptom

Key: *Ss* total sample size, *f* female, *m* male, *n* number, *NRS* Numerical rating scale, *BMI* Body Mass Index, *kg* kilogram, *m2* meter square, *ESWT* Extracorporeal shockwave therapy, *VAS* Visual Analog Scale, *FFI* Foot Function Index, *GROC* Global Rating of Change, *PSFS* Patient Specific functional scale, *PF* Plantar Flexion, *IR* Internal Rotation, *ER* External Rotation

### Quality assessment

The quality of four studies [29–32] were evaluated using the QUADCPR [20] and one single cohort study by Hansen et al. (2018) [9] was evaluated using the EAI tool [21]. The reporting of the study aims, setting and description of sample characteristics were found to be of good quality. However, there were a lack of information regarding reliability and validity of the main outcome measures used, first order interaction in the statistical analyses, validity and reliability of the model, and covariate/confounders analyses for the factors according to EAI. The items which led to discrepancy between two reviewers were “outcome measure reliability, validity and sensitivity to change”, “first order interactions were assessed and reported” and “irrelevant predictors removed prior to multivariate modelling” in the QUADCPR assessment tool. Details of the quality assessments are presented in Table 2.

### Risk of bias assessment

**Fig. 3:**
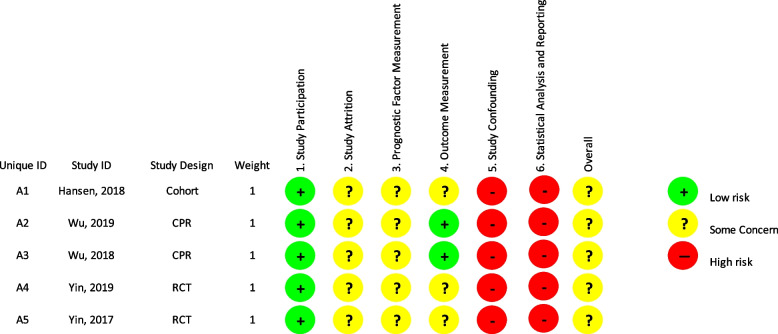
Risk of Bias assessment using QUIPS II; Key: CPR = Clinical prediction rule, RCT = randomized controlled trial

There were 30 domains in total across the five studies, with 7 domains (23%) classed as low, 13 (43%) classed as moderate, and 10 (34%) classed as high RoB (Fig. 3) [29, 30]. There were no missing data for prognostic factor measurements in four studies. However, Hansen et al. did not report in the data analysis section if the study had any missing/incomplete data due to drop-out [9]. For outcome measurements, three studies [9, 31, 32] were classified as having moderate RoB because specific clinical or imaging outcome measurements were either inadequately described or not stated [9, 31, 32]. Regarding the study confounding domain, all studies were scored as having a high RoB because definitions of confounding factors or adjustments were either unclear or not reported. Finally, all studies had moderate RoB on the statistical analysis domain as data were presented with insufficient detail, with the justification for statistical modelling outlined but no evidence of selective reporting.

**Table 4:**
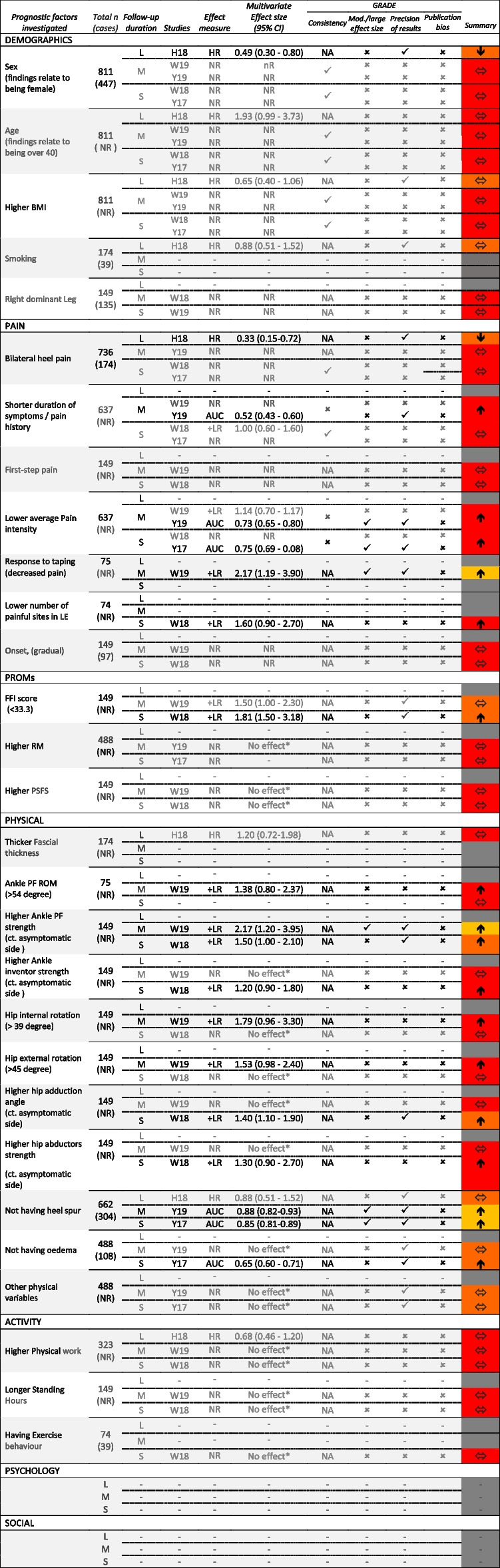
Investigated prognostic factors across long-, medium- and short-term follow-up duration, with effect measure, size, direction and GRADE which is coded using colour system in the last column. Red, orange, yellow and green show very low, low, moderate, and high-level of evidence, respectively. Grey is no investigation/evidence in relevant

KEY = -: not investigated; Results in BOLD type reveal a statistically significant results (*p* < 0.05). Case refers to number of variables which have been indicated to be a predictive factors

*NA* not applicable, *HR* Hazard Ratio, *RR* Relative Risk, *AUC* Area under the curve, + *LR* positive likelihood ratio, *NR* Not Reported, *L* Long Term, *M* Medium term, *S* Short-term outcomes

^*^No reported effect, studies provided only p value. Ct: Compare to. Articles: H18: Hansen et. al, 2018 (Cohort study); W18: Wu et. al, 2018 (RCTs); W19: Wu et. al, 2019 (RCTs); Y17: Yin et. al, 2017 (RCTs); Y19: Yin et. al, 2019 (RCTs). Red, orange, yellow and green show very low, low, moderate, and high-level of evidence, respectively. Grey is no investigation/evidence in the relevant period. Arrow key: Up arrow: the value of the factor has a positive effect on prognosis; down arrow: the value of the factor has a negative effect on prognosis; Horizontal arrow: Prognosis probability is not affected by a change in the value. Publication bias and study limitation of GRADE’s domains are not shown in the table due to similar results across all studies (i.e. negative). Regarding the precision of studies, studies scored as unclear—not having SD or CI are considered as imprecise. For GRADE factors: ✓, no serious limitations; ✕, serious limitations (or not present for moderate/large effect size, dose effect); unclear, unable to rate item based on available information

### Summary of findings

Studies in this review reported two directions (favourable vs unfavourable) of a statistically significant relationship. All estimate sizes of the relationships were presented as reported in the source multivariate analyses. Included below are a summary of findings presented under two headings including (i) participant characteristics associated with an outcome in a cohort study and (ii) participant characteristics associated with an outcome after a specific treatment.

### Participant characteristics associated with an outcome in a cohort study

One study investigated the association between participants’ baseline characteristics and a poor PHP outcome [9]. Ninety-three percent of participants in this cohort study were reported to receive various treatment strategies such as injections, insoles, exercises and ESWT. Nine patient-reported and anatomical characteristics were investigated. Multiple Cox regression analyses revealed only two patient characteristics (sex and having bilateral heel pain) were associated with a poor outcome (Table 3).

#### Demographics

There was low evidence of a small effect that a patient being female was a predictor of an unfavourable outcome in the long term (HR: 0.49 [0.30–0.80]) (i.e. “for every 100 men cured per year, only 49 women were cured”) [9]. BMI and smoking were not shown to be significant prognostic factors at this time-point (Table 4).

#### Pain-related factors

There was low evidence of a small effect that having bilateral heel pain was a predictor of an unfavourable outcome in the long term of PHP when controlling for sex, age BMI, smoking, physical work, time to ultrasound, fascia thickness and heel spur. The hazard ratio of 0.33 [0.15–0.72] indicates that the chance of being asymptomatic for those with bilateral pain was 33% relative to people with unilateral pain [9]. It should be acknowledged that being asymptomatic for those with bilateral pain referred to pain relief on the most affected side.

### Participant characteristics associated with an outcome after a specific treatment

Three different specific treatments, foot orthoses [30], biomechanical anti-pronation taping [29] and extracorporeal shock wave therapy (ESWT) were investigated in four studies [29–32]. Two studies investigated predictive factors for a minimum clinically successful therapy after extracorporeal shock wave therapy at 3 and 6 months [31, 32]. Fourteen participant characteristics, including pain, physical and function-related factors, were reported to be associated with a successful outcome after a specific treatment.

#### Pain-related factors

There is very low evidence of small effect that the number of painful sites in the lower back and the lower extremity region were a predictor of success for anti-pronation taping intervention in the short term [29]. Authors included the number of painful sites as a potential independent variable in their prognostic models recognising the potential the biomechanical interaction between foot pronation and lower extremity pathologies. Similarly, there is moderate evidence of a large effect that decreased pain by over 1.5 points (on a 10 point scale), as a response to anti-pronation taping, was a predictor of foot orthoses success in the medium term when controlling for range of ankle plantar flexion, ankle plantar-flexor strength, and range of hip internal–external rotation (+ LR: 2.17 [1.19–3.90]) [30] (Table 4). The indicator of foot orthoses success and study characteristics of the other four included publications were presented in Table 3.

There was very low evidence of small effect that a shorter history of symptoms and average pain intensity predicted a favourable outcome following an ESWT intervention in the medium term when controlling for the presence of a heel spur (AUC: 0.52 [0.43–0.6], 0.73 [0.65–0.80], respectively) [32]. Average pain intensity was also a predictor of a favourable outcome in the short term, for the same intervention, when controlling for the presence of oedema and a heel spur (AUC: 0.75 [0.69–0.08]) [31]. There were no associations found between PHP prognosis and either bilateral heel pain, first step pain or the onset of pain (P value > 0.05).

#### Patient Reported Outcome Measures (PROMs)

There was low evidence of a small effect that scoring lower than 33.3 on the FFI was a predictor of anti-pronation taping intervention success in the short term (+ LR: 1.81 [1.50–3.18]) [29]. Results revealed no significant evidence for the predictive effects of the PSFS and Roles and Maudsley score (RM) on the prognosis of PHP or a favourable outcome to an intervention (Table 4).

#### Physical factors

There was very low evidence of small effect that increased ankle plantar flexor ROM (> 54°), reduced hip internal ROM (< 39°) and increased hip external rotation (> 45°) were positive predictors of foot orthoses intervention success in the medium term (+ LR: 1.38 [0.80–2.37], 1.79 [0.96–3.30], 1.53 [0.98–2.40], respectively) [30]. However, it is important to note that the LR values where the associated 95% confidence intervals contain 1 suggest that these the values were not precise enough to be statistically significant although they were indicated as meaningful predictors in the source paper. In addition to this, there was low evidence of a small effect that higher or equal ankle plantar flexor strength compared to the asymptomatic side predicted a favourable outcome of foot orthoses in the medium term (+ LR: 2.17 [1.20–3.95]) [30]. However, there was only low evidence of a small effect size that the plantar flexor strength variable was a positive predictor of the favourable outcome of anti-pronation taping in the short term (+ LR: 1.50 [1.00- 2.10]) [29]. There was low evidence of a small effect that greater hip adduction angle in the symptomatic side was a positive predictor of anti-pronation taping intervention success in the short term (+ LR: 1.40 [1.10–1.90]) [29]. There was very low evidence of a small effect that lower ankle invertor, hip abductors and ankle plantar flexion (PF) strength in the symptomatic side were positive predictors of favourable outcome for the anti-pronation taping intervention in the short term (+ LR: 1.20 [0.90–1.80], 1.30 [0.90–2.70], respectively) [29].

There was low evidence of a small effect that not having oedema was an indicator of a favourable outcome of ESWT intervention in the short term (AUC: 0.65 [0.60–0.71]) (Table 4) [31]. Finally, there was moderate evidence of a large effect that not having a heel spur predicted a favourable outcome of ESWT in the short to medium term when controlling for oedema and average pain intensity (AUC: 0.88 [0.82–0.93], 0.85 [0.81–0.89], respectively) [31, 32]. There were also other lower extremity variables (details are reported in the supplementary file) that were tested, however, none of them were found as statistically significant predictors of PHP prognosis (P values > 0.05).

#### Activity

Results revealed that physical work was not associated with an outcome for ESWT, anti-pronation taping or an orthotic intervention in the short and medium term. Standing hours and exercise behaviour were not associated with the outcome for anti-pronation taping and foot orthoses in the short and medium term (Table 4).

## Discussion

This systematic review aimed to provide a comprehensive examination of patient characteristics associated with outcomes from 811 people with PHP. We found that people with PHP who are female and have bilateral heel pain are at risk of a poor outcome as revealed by a single prospective cohort study [9]. The immediate effects of taping, symptom duration and the number of painful sites are also prognostic indicators of recovery, as are a variety of ankle and hip kinematics such as increased ankle plantar flexion and hip rotation range. However, it is important to note that those prognostic factors are for PHP in the context of specific interventions such as anti-pronation taping, orthosis and shockwave therapy and therefore not generalisable. There is a major need for high quality, detailed, adequately powered prospective studies of prognostic factors. These should cover a range of biopsychosocial domains for this common, problematic and recalcitrant condition.

The single cohort study by Hansen et al., (2018) [9] revealed that having bilateral heel pain and being female were predictive of a negative prognosis. Several studies report that sex differences related to pain and recovery exist [33, 34]. However, the specific underlying mechanisms contributing to this disparity are unknown. Therefore, further research exploring the effect of sex on recovery of PHP is needed and earlier interventions might need to be considered to prevent chronicity for female patients.

Hansen et al. (2018) [9] reported a poorer outcome for patients with bilateral heel pain. People with PHP usually develop PHP in a single foot initially [35–37] with symptoms becoming present in the contralateral foot as severity increases, possibly due to altered gait or because intrinsic and extrinsic risk factors apply to both limbs. Further, those with bilateral symptoms are likely to be more severely affected [38], which is important to consider for planning management.

In a clinical prediction rules study that reported the short-term use (2–3 days) of anti-pronation taping, it was revealed that the number of painful sites in the lower back and lower extremity regions were associated with a favourable outcome for anti-pronation taping. Therefore, clinicians might consider assessing the total number of painful sites -particularly the low limb and low back- to understand the severity of plantar heel pain.

Wu et al. (2018) who reported clinical prediction rules for anti-pronation taping revealed that various physical factors were associated with a favourable outcome [29]. These included having ankle plantar flexor and hip abduction strength equal or greater than the contra-lateral side, weaker ankle inverters and a greater range of hip adduction. It is not possible to determine that these factors are causative from this paper. However, it could be postulated that subjects with better strength and flexibility at the hip, with a bias to weak ankle inversion, have a better response to anti-pronation taping.

Prognostic research for PHP would be improved by the development of a PROM that is better suited to the particular presentation of PHP. The FFI score was identified as a PROM that predicted success of anti-pronation taping by measuring functional severity at baseline (i.e. There was low evidence of a small effect that scoring lower than 33.3 on the FFI was a predictor of anti-pronation taping intervention success in the short term) [29]. There are also other PROMs with better responsiveness for people with PHP such as the Foot Health Status Questionnaire and Foot and Ankle Ability Measure [17, 39]. However, it is important to note that all of these lack a question about first step pain which is pathognomonic for PHP and is the most prominent symptom. Developing a PROM that accurately captures the essence of the difficulties faced by people with PHP should facilitate understanding of the condition, including prognosis.

Wu et al. (2019) [30] reported that when the average pain intensity decreased by over 1.5 points with anti-pronation taping, it was associated with a favourable outcome for an orthoses intervention. Intuitively, the benefit of taping that mimics orthoses would seem logical because both interventions provide a biomechanical support on the foot to decrease stress on the plantar fascia [40, 41]. Taking into account that this factor has a high effect size (+ LR = 2.17 (1.19–3.90)), and anti-pronation taping is more feasible compared to orthoses applications in the first line management strategies (at a time period of 1 week), clinicians are recommended to apply taping to people who are potentially eligible for foot orthoses prescription.

The results revealed by Wu et al. (2019) [30] suggest that increased ankle plantar flexion, hip internal and external rotation angle are associated with a positive outcome from the use of foot orthoses [30]. With reference to the predictors relating to hip mobility and ankle plantar flexion, it has been postulated that these variables might be associated with an out-toeing gait, leading to an increase in medial tibial rotation and excessive foot pronation [42], which could also be targeted and controlled with foot orthoses [43]. The importance of these factors is likely driven by the key role of the plantar fascia in gait and its anatomical location meaning there are high magnitude compressive and tensile forces acting on the tissue. The degree and importance of these physical outcome predictors requires clarification and confirmation in future studies.

A shorter symptom duration with a lower frequency of pain was reported to be predictive of a favourable outcome following ESWT. However, it is important to note that the AUC value is close to the threshold of 0.50 which suggests no relevant relationship or no ability of this factor to discriminate between those with an unfavourable or favourable outcome with ESWT. Similarly, higher pain severity at baseline and longer pain duration have also shown an association with a poor prognosis in other musculoskeletal pain conditions [44]. Irrespective of the type of treatment strategy implemented for a musculoskeletal condition, these findings highlight the clinical importance of implementing an effective pain intervention programme as early as possible in order to increase the likelihood of intervention success.

The absence of research on prognosis related to the role of psychosocial factors for people with PHP is an area where further research is needed. There is a substantial body of evidence that psychological disorders are associated with bodily pain in several musculoskeletal conditions including PHP [45–47]. Of these, emotional (e.g., depression), cognitive (e.g. catastrophisation) and behavioural (e.g. avoidance behaviours) factors have received the most attention within several case–control and cross-sectional studies [48–51]. Understanding the role of psychosocial aspects of a musculoskeletal condition will assist management strategies [11].

Additionally, developing prognostic models is a process with several steps; starting from evaluation of prognostic factors, followed by model development and validation [52, 53]. It should be emphasised that the current evidence base is relevant only to the initial stage of prognostic research, with no work yet reporting validation of a prognostic model. Therefore, second and third phase prognostic studies are clearly needed to inform clinical practice.

The most commonly found limitation across the studies was inadequate reporting of statistical and methodological approaches. These studies also did not provide estimate rates from the univariate analyses, which limited interpretation of the individual association of variables on prognosis. It is recommended that authors apply published recommendations, designed to improve the quality and transparency of prognosis research [54]. Moreover, it has been critiqued that Wu et al. (2018) [29] tested only one version of anti-pronation taping without dynamic gait analysis and the benefit of implementation of this tape or a different version in the longer term is unknown. The same group also evaluated the outcome of orthoses [30]. Although orthoses are termed customised, the authors utilised heat-mouldable preformed orthoses and the method of customisation was not described. Additionally, it was not clearly stated if the results from Wu et al. (2018) [29] and Wu et al. (2019) [30] were from the same sample.

There were limitations of the review process. First, relevant databases were thoroughly searched using keywords, but there is always the risk of missing relevant studies, particularly for single arms of intervention trials. In order to avoid missing any study, we performed double screening of RCT arms through the search returns of our recent systematic review which had sourced all RCTs of any intervention in any language [6]. Moreover, both reviewers were blinded to the authors of the papers included for appraisal [23]. Second, although this systematic review only implemented a narrative synthesis, variations in recovery definition (Table 3) could influence the interpretation of estimates summarised across the retained studies. Therefore, these limitations should be considered during interpretation of the results. Third, previous research has used either QUADCPR or QUIPS II for the quality assessment. However, as Butner et al [23] points out, the former tool assesses methodological quality of studies whereas the latter is focusing on risk of bias. In this study, we used both QUADCPR and QUIPS II.

## Conclusion

There are limited biomedical factors which can be used to predict PHP outcomes. Having bilateral pain and being female should alert clinicians to an increased risk of a poor outcome. We identified modifiable and measurable factors such as pain status and a variety of ankle and hip kinematics, as being potential factors that predict the success of treatments such as shockwave therapy, anti-pronation taping and foot orthoses. These could assist informed clinical decisions regarding outcome expectations. To better understand PHP recovery or persistence, high quality prospective studies should evaluate the prognostic value of a range of variables, including psychosocial in addition to biological factors.

## Supplementary Information

**Additional file 1.**

## Data Availability

The datasets analysed during the current study are available from the corresponding author on reasonable request.

## Abbreviations

RCT: Randomized controlled trial
ESWT: Extracorporeal shockwave therapy
ROB: Risk of bias
ROM: Range of motion
FFI: Foot function index
AUC: Area under the curve
LR: Likelihood ratio
HR: Hazard ratio
BMI: Body mass index
MLA: Medial longitudinal arch
MTPJ1: First metatarsophalangeal joint
PHP: Plantar heel pain
PROMs: Patient reported outcome measures

## References

[1] Dunn J, Link C, Felson D, Crincoli M, Keysor J, McKinlay J (2004). Prevalence of foot and ankle conditions in a multiethnic community sample of older adults. Am J Epidemiol.

[2] Hill CL, Gill TK, Menz HB, Taylor AW (2008). Prevalence and correlates of foot pain in a population-based study: the North West Adelaide health study. J Foot Ankle Res.

[3] Menz HB, Tiedemann A, Kwan M, Plumb K, Lord SR (2006). Foot pain in community-dwelling older people: an evaluation of the Manchester foot pain and disability index. Rheumatology.

[4] Thomas MJ, Whittle R, Menz HB, Rathod-Mistry T, Marshall M, Roddy E (2019). Plantar heel pain in middle-aged and older adults: population prevalence, associations with health status and lifestyle factors, and frequency of healthcare use. BMC Musculoskelet Disord.

[5] Irving DB, Cook JL, Young MA, Menz HB (2008). Impact of chronic plantar heel pain on health-related quality of life. J Am Podiatr Med Assoc.

[6] Morrissey D, Cotchett M, J'Bari AS, Prior T, Griffiths IB, Rathleff MS (2021). Management of plantar heel pain: a best practice guide informed by a systematic review, expert clinical reasoning and patient values. Br J Sports Med.

[7] Radford JA, Landorf KB, Buchbinder R, Cook C (2007). Effectiveness of calf muscle stretching for the short-term treatment of plantar heel pain: a randomised trial. BMC Musculoskelet Disord.

[8] Salvioli S, Guidi M, Marcotulli G (2017). The effectiveness of conservative, non-pharmacological treatment, of plantar heel pain: a systematic review with meta-analysis. Foot.

[9] Hansen L, Krogh TP, Ellingsen T, Bolvig L, Fredberg U (2018). Long-term prognosis of plantar fasciitis: a 5-to 15-year follow-up study of 174 patients with ultrasound examination. Orthop J Sport Med.

[10] Collins NJ, Bierma-Zeinstra SM, Crossley KM, van Linschoten RL, Vicenzino B, van Middelkoop M (2013). Prognostic factors for patellofemoral pain: a multicentre observational analysis. Br J Sports Med.

[11] Mallows A, Debenham J, Walker T, Littlewood C (2017). Association of psychological variables and outcome in tendinopathy: a systematic review. Br J Sports Med.

[12] Hutton B, Salanti G, Caldwell DM, Chaimani A, Schmid CH, Cameron C (2015). The PRISMA extension statement for reporting of systematic reviews incorporating network meta-analyses of health care interventions: checklist and explanations. Ann Intern Med.

[13] Riley RD, Moons KG, Snell KI, Ensor J, Hooft L, Altman DG (2019). A guide to systematic review and meta-analysis of prognostic factor studies. BMJ.

[14] Van Leeuwen K, Rogers J, Winzenberg T, van Middelkoop M (2016). Higher body mass index is associated with plantar fasciopathy/‘plantar fasciitis’: systematic review and meta-analysis of various clinical and imaging risk factors. Br J Sports Med.

[15] Matthews M, Rathleff MS, Claus A, McPoil T, Nee R, Crossley K (2017). Can we predict the outcome for people with patellofemoral pain? a systematic review on prognostic factors and treatment effect modifiers. Br J Sports Med.

[16] Shekelle PG, Maglione MA, Luoto J, Johnsen B, Perry TR. Using six different frameworks to assess the evidence for three examples of health interventions or programs. In: Global health evidence evaluation framework. Agency for Healthcare Research and Quality (US); 2013.

[17] Martin RL, Davenport TE, Reischl SF, McPoil TG, Matheson JW, Wukich DK (2014). Heel pain—plantar fasciitis: revision 2014. J Orthop Sports Phys Ther.

[18] Moons KG, de Groot JA, Bouwmeester W, Vergouwe Y, Mallett S, Altman DG (2014). Critical appraisal and data extraction for systematic reviews of prediction modelling studies: the CHARMS checklist. PLoS Med.

[19] Wade DT, Halligan PW (2017). The biopsychosocial model of illness: a model whose time has come. Clin Rehabil..

[20] Cook C, Brismée J-M, Pietrobon R, Sizer P, Hegedus E, Riddle DL (2010). Development of a quality checklist using Delphi methods for prescriptive clinical prediction rules: the QUADCPR. J Manipulative Physiol Ther.

[21] Genaidy A, Lemasters G, Lockey J, Succop P, Deddens J, Sobeih T (2007). An epidemiological appraisal instrument–a tool for evaluation of epidemiological studies. Ergonomics.

[22] Hayden JA, van der Windt DA, Cartwright JL, Côté P, Bombardier C (2013). Assessing bias in studies of prognostic factors. Ann Intern Med.

[23] Büttner F, Winters M, Delahunt E, Elbers R, Lura CB, Khan KM (2020). Identifying the ‘incredible’! Part 2: Spot the difference - a rigorous risk of bias assessment can alter the main findings of a systematic review. Br J Sports Med.

[24] Huguet A, Hayden JA, Stinson J, McGrath PJ, Chambers CT, Tougas ME (2013). Judging the quality of evidence in reviews of prognostic factor research: adapting the GRADE framework. Syst Rev.

[25] Iorio A, Spencer FA, Falavigna M, Alba C, Lang E, Burnand B, et al. Use of GRADE for assessment of evidence about prognosis: rating confidence in estimates of event rates in broad categories of patients. BMJ. 2015;350:h870. 10.1136/bmj.h870 25775931

[26] Azuero A (2016). A note on the magnitude of hazard ratios. Cancer.

[27] Rubinstein ML, Kraft CS, Parrott JS (2018). Determining qualitative effect size ratings using a likelihood ratio scatter matrix in diagnostic test accuracy systematic reviews. Diagnosis.

[28] Menard S (2002). Applied logistic regression analysis.

[29] Wu F-L, Shih Y-F, Lee S-H, Luo H-J, Wang WT-J (2018). Development of a clinical prediction rule to identify patients with plantar heel pain likely to benefit from biomechanical anti-pronation taping: a prospective cohort study. Phys Ther Sport.

[30] Wu F-L, Shih Y-F, Lee S-H, Luo H-J, Wang WT-J (2019). Can short-term effectiveness of anti-pronation taping predict the long-term outcomes of customized foot orthoses: developing predictors to identify characteristics of patients with plantar heel pain likely to benefit from customized foot orthoses. BMC Musculoskelet Disord.

[31] Yin M, Chen N, Huang Q, Marla AS, Ma J, Ye J (2017). New and accurate predictive model for the efficacy of extracorporeal shock wave therapy in managing patients with chronic plantar fasciitis. Arch Phys Med Rehabil.

[32] Yin M, Ma J, Xu J, Li L, Chen G, Sun Z (2019). Use of artificial neural networks to identify the predictive factors of extracorporeal shock wave therapy treating patients with chronic plantar fasciitis. Sci Rep.

[33] Macaluso A, De Vito G (2004). Muscle strength, power and adaptations to resistance training in older people. Eur J Appl Physiol.

[34] Mitchell W, Atherton P, Williams J, Larvin M, Lund J, Narici M (2012). Sarcopenia, Dynapenia, and the Impact of Advancing Age on Human Skeletal Muscle Size and Strength; a Quantitative Review. Front Physiol.

[35] Beeson P (2014). Plantar fasciopathy: revisiting the risk factors. Foot Ankle Surg.

[36] Irving DB, Cook JL, Young MA, Menz HB (2007). Obesity and pronated foot type may increase the risk of chronic plantar heel pain: a matched case-control study. BMC Musculoskelet Disord.

[37] Irving D, Cook J, Menz H (2006). Factors associated with chronic plantar heel pain: a matched case–control study. J Sci Med Sport.

[38] Phillips A, McClinton S (2017). Gait deviations associated with plantar heel pain: a systematic review. Clin Biomech.

[39] Martin RL, Irrgang JJ (2007). A survey of self-reported outcome instruments for the foot and ankle. J Orthop Sports Phys Ther.

[40] Meier K, McPoil TG, Cornwall MW, Lyle T (2008). Use of antipronation taping to determine foot orthoses prescription: a case series. Res Sports Med.

[41] Vicenzino B (2004). Foot orthotics in the treatment of lower limb conditions: a musculoskeletal physiotherapy perspective. Man Ther.

[42] Resende RA, Deluzio KJ, Kirkwood RN, Hassan EA, Fonseca ST (2015). Increased unilateral foot pronation affects lower limbs and pelvic biomechanics during walking. Gait Posture.

[43] Lee MS, Vanore JV, Thomas JL, Catanzariti AR, Kogler G, Kravitz SR (2005). Diagnosis and treatment of adult flatfoot. J Foot Ankle Surg.

[44] Artus M, Campbell P, Mallen CD, Dunn KM, van der Windt DAW (2017). Generic prognostic factors for musculoskeletal pain in primary care: a systematic review. BMJ Open.

[45] Woolf AD, Erwin J, March L (2012). The need to address the burden of musculoskeletal conditions. Best Pract Res Clin Rheumatol.

[46] Cimmino MA, Ferrone C, Cutolo M (2011). Epidemiology of chronic musculoskeletal pain. Best Pract Res Clin Rheumatol.

[47] Crofford LJ (2015). Psychological aspects of chronic musculoskeletal pain. Best Pract Res Clin Rheumatol.

[48] Chester R, Jerosch-Herold C, Lewis J, Shepstone L (2018). Psychological factors are associated with the outcome of physiotherapy for people with shoulder pain: a multicentre longitudinal cohort study. Br J Sports Med.

[49] Cotchett M, Lennecke A, Medica VG, Whittaker GA, Bonanno DR (2017). The association between pain catastrophising and kinesiophobia with pain and function in people with plantar heel pain. Foot.

[50] Cotchett M, Munteanu SE, Landorf KB (2016). Depression, anxiety, and stress in people with and without plantar heel pain. Foot Ankle Int.

[51] Cotchett MP, Whittaker G, Erbas B (2015). Psychological variables associated with foot function and foot pain in patients with plantar heel pain. Clin Rheumatol.

[52] Steyerberg EW, Moons KGM, van der Windt DA, Hayden JA, Perel P, Schroter S (2013). Prognosis Research Strategy (PROGRESS) 3: prognostic model research. PLoS Med.

[53] Riley RD, Hayden JA, Steyerberg EW, Moons KGM, Abrams K, Kyzas PA (2013). Prognosis Research Strategy (PROGRESS) 2: prognostic factor research. PLoS Med.

[54] Riley RD, Moons KGM, Snell KIE, Ensor J, Hooft L, Altman DG (2019). A guide to systematic review and meta-analysis of prognostic factor studies. BMJ.

