# Supplementary material for: Predicting the outcome of plantar heel pain in adults: a systematic review of prognostic factors
Source: J Foot Ankle Res. 2023 May 12;16:28. doi: 10.1186/s13047-023-00626-y (PMC10176769; doi:10.1186/s13047-023-00626-y)
Supplement: Supplementary file 1 — Additional file 1. [file 13047_2023_626_MOESM1_ESM.docx]

# SUPPLEMENTARY FILE

## 1. Search Strategy

| Search Terms | | |
| --- | --- | --- |
| Construct | | Keywords |
| Participants | AND  [tiab] | Painful-heel-syndrome OR plantar-fasciitis OR plantar-fasciopathy OR subcalcaneal-bursitis OR medial-arch-pain OR subcalcaneal-pain OR stone-bruise OR calcaneal-periostitis OR subcalcaneal-spur OR calcaneodynia OR Heel-Spur-Syndrome OR Chronic-Plantar-Fasciitis OR Fasciitis-Chronic-Plantar OR Plantar-Fasciitis-Chronic OR Fasciitis-Plantar-Chronic OR plantar-heel-pain |
| Factors/variables | AND  [tiab] | Predict* OR prognosis* OR prognostic OR indicat* OR disease course OR disease progression OR follow-up OR natural history OR factor* OR associated factor* |
| Study type | AND  [tiab] | Observational OR cohort OR prospective OR case-control OR Longitudin* OR randomised controlled OR randomized controlled OR randomised clinical OR randomized clinical |
| Study type | NOT [ti] | retrospective OR cross AND sectional OR systematic AND review OR literature AND review OR scoping AND review OR meta AND analysis |

## 2. Database search

Embase: 319

('painful heel syndrome':ab,ti OR 'plantar fasciitis':ab,ti OR 'plantar fasciopathy':ab,ti OR 'subcalcaneal bursitis':ab,ti OR 'medial arch pain':ab,ti OR 'subcalcaneal pain':ab,ti OR 'stone bruise':ab,ti OR 'calcaneal periostitis':ab,ti OR 'subcalcaneal spur':ab,ti OR calcaneodynia:ab,ti OR 'heel spur syndrome':ab,ti OR 'chronic plantar fasciitis':ab,ti OR 'fasciitis chronic plantar':ab,ti OR 'plantar fasciitis chronic':ab,ti OR 'fasciitis plantar chronic':ab,ti OR 'plantar heel pain':ab,ti) AND (predict*:ab,ti OR prognosis*:ab,ti OR prognostic:ab,ti OR indicat*:ab,ti OR 'disease course':ab,ti OR 'disease progression':ab,ti OR 'follow up':ab,ti OR 'natural history':ab,ti OR factor*:ab,ti OR 'risk factor*':ab,ti OR 'associated factor*':ab,ti) AND (observational:ab,ti OR cohort:ab,ti OR prospective:ab,ti OR 'case control':ab,ti OR longitudinal:ab,ti OR 'randomised controlled':ab,ti OR 'randomized controlled':ab,ti OR 'randomised clinical':ab,ti OR 'randomized clinical':ab,ti) NOT ((((((retrospective:ti OR cross:ti) AND sectional:ti OR systematic:ti) AND review:ti OR literature:ti) AND review:ti OR scoping:ti) AND review:ti OR meta:ti) AND analysis:ti)

Medline OvidSP: 586

(((Painful-heel-syndrome OR plantar-fasciitis OR plantar-fasciopathy OR subcalcaneal-bursitis OR medial-arch-pain OR subcalcaneal-pain OR stone-bruise OR calcaneal-periostitis OR subcalcaneal-spur OR calcaneodynia OR Heel-Spur-Syndrome OR Chronic-Plantar-Fasciitis OR Fasciitis-Chronic-Plantar OR Plantar-Fasciitis-Chronic OR Fasciitis-Plantar-Chronic OR plantar-heel-pain) AND (Predict* OR prognosis* OR prognostic OR indicat* OR disease course OR disease progression OR follow-up OR natural history OR factor* OR risk factor* OR associated factor* ) AND (Observational OR cohort OR prospective OR case-control OR Longitudinal OR randomised controlled OR randomized controlled OR randomised clinical OR randomized clinical )).ab,ti.) NOT ((retrospective OR cross AND sectional OR systematic AND review OR literature AND review OR scoping AND review OR Meta AND analyses).ti.)

Web-of-Science: 304

TS=((Painful-heel-syndrome OR plantar-fasciitis OR plantar-fasciopathy OR subcalcaneal-bursitis OR medial-arch-pain OR subcalcaneal-pain OR stone-bruise OR calcaneal-periostitis OR subcalcaneal-spur OR calcaneodynia OR Heel-Spur-Syndrome OR Chronic-Plantar-Fasciitis OR Fasciitis-Chronic-Plantar OR Plantar-Fasciitis-Chronic OR Fasciitis-Plantar-Chronic OR plantar-heel-pain) AND (Predict* OR prognosis* OR prognostic OR indicat* OR disease course OR disease progression OR follow-up OR natural history OR factor* OR risk factor* OR associated factor* ) AND (Observational OR cohort OR prospective OR case-control OR Longitudinal OR randomised controlled OR randomized controlled OR randomised clinical OR randomized clinical )) NOT TI=(retrospective OR cross AND sectional OR systematic AND review OR literature AND review OR scoping AND review OR meta AND analyses )

Pubmed: 520

(((Painful-heel-syndrome[Title/Abstract] OR plantar-fasciitis[Title/Abstract] OR plantar-fasciopathy[Title/Abstract] OR subcalcaneal-bursitis[Title/Abstract] OR medial-arch-pain[Title/Abstract] OR subcalcaneal-pain[Title/Abstract] OR stone-bruise[Title/Abstract] OR calcaneal-periostitis[Title/Abstract] OR subcalcaneal-spur[Title/Abstract] OR calcaneodynia[Title/Abstract] OR Heel-Spur-Syndrome[Title/Abstract] OR Chronic-Plantar-Fasciitis[Title/Abstract] OR Fasciitis-Chronic-Plantar[Title/Abstract] OR Plantar-Fasciitis-Chronic[Title/Abstract] OR Fasciitis-Plantar-Chronic[Title/Abstract] OR plantar-heel-pain[Title/Abstract])) AND (Observational[Title/Abstract] OR cohort[Title/Abstract] OR prospective[Title/Abstract] OR case-control[Title/Abstract] OR Longitudinal[Title/Abstract] OR randomised controlled[Title/Abstract] OR randomized controlled[Title/Abstract] OR randomised Clinical[Title/Abstract] OR randomized clinical[Title/Abstract])) NOT (NOT retrospective NOT cross-sectional NOT systematic review [Title])

Scopus: 21

( TITLE-ABS-KEY ( painful-heel-syndrome OR plantar-fasciitis OR plantar-fasciopathy OR subcalcaneal-bursitis OR medial-arch-pain OR subcalcaneal-pain OR stone-bruise OR calcaneal-periostitis OR subcalcaneal-spur OR calcaneodynia OR heel-spur-syndrome OR chronic-plantar-fasc ) AND TITLE-ABS-KEY ( predict* OR prognosis* OR prognostic OR indicat* OR disease AND course OR disease AND progression OR follow-up OR natural AND history OR factor* OR risk AND factor* OR associated AND factor* ) AND TITLE-ABS-KEY ( observational OR cohort OR prospective OR case-control OR longitudinal OR randomised AND controlled OR randomized AND controlled OR randomised AND clinical OR randomized AND clinical ) AND NOT TITLE ( retrospective OR cross AND sectional OR systematic AND review OR literature AND review OR scoping AND review OR meta AND analysis ) )

## 3. Determining range of effect size measures based on previous literature

Small, medium, and large HRs for a standard deviation increase in the predictor were 1.14, 1.47, and 1.9, respectively.(1) LR+ 5–10 generate moderate probability; LR+ 2–5 generate small but important probability; LR+ 1–2 generate small but rarely important probability.(2) Regarding the AUC, a ROC = 0.5, suggests no discrimination; 0.7 < ROC < 0.8 is considered acceptable; 0.8 < ROC <0.9 is considered excellent and if the ROC > 0.9 it is considered outstanding.(3)

## 4. Outcome measures investigating in the reviewed articles

| Hansen et al, 2018 | Age, gender, Body mass index, onset of symptoms, Physical work, Smoking, Exercise-induced symptoms, Time from onset of symptoms to baseline, Duration of symptoms, Number of treatments tried, relapse |
| --- | --- |
| Wu et al, 2018 | Age, gender, Body mass index, Duration of symptom, First-step pain, Usual pain, PSFS, FFI, Weekly working time, Daily weight-bearing time, Dominant leg, Mode of onset, Episode, Rearfoot varus in STNP, Forefoot varus in STNP, Rearfoot inversion ROM, Rearfoot eversion ROM, 1st MTP joint extension ROM, Ankle dorsiflexion ROM, Ankle plantarflexion ROM, Navicular drop test, Calcaneus valgus in RCSP, Calcaneus valgus in NCSP, Foot posture index, Femoral neck anteversion, Tibial lateral torsion, Leg length difference, Quadriceps angle, Hip internal rotation ROM, Hip external rotation ROM, Straight leg raise range, Hip flexion ROM in Thomas test, Knee flexion ROM in Thomas test, Hip adduction ROM in Ober’s test, Ankle dorsiflexor, Ankle plantarflexor, Ankle invertor, Ankle evertor, 1st toe flexor, Knee flexor, Knee extensor, Hip flexor, Hip extensor, Hip abductor |
| Wu et al, 2019 | Age, gender, Body mass index, Duration of symptom, First-step pain, Usual pain, PSFS, FFI, Weekly working time, Daily weight-bearing time, Without exercise behaviour, Dominant leg, Mode of onset, Episode, With bilateral plantar heel pain, Number of painful sites in the low back and lower extremity regions, Rearfoot varus in STNP, Forefoot varus in STNP, Rearfoot inversion ROM, Rearfoot eversion ROM, 1st MTP joint extension ROM, Ankle dorsiflexion ROM, Ankle plantarflexion ROM, Navicular drop test, Calcaneus valgus in RCSP, Calcaneus valgus in NCSP, Foot posture index, Femoral neck anteversion, Tibial lateral torsion, Leg length difference, Quadriceps angle, Hip internal rotation ROM, Hip external rotation ROM, Straight leg raise range, Hip flexion ROM in Thomas test, Knee flexion ROM in Thomas test, Hip adduction ROM in Ober’s test, Tibial varum angle in standing, Ankle dorsiflexor, Ankle plantarflexor, Ankle invertor, Ankle evertor, 1st toe flexor, Knee flexor, Knee extensor, Hip flexor, Hip extensor, Hip abductor |
| Yin et al 2017 | Age (years), Gender, Body mass index, affected bilateral side, duration of symptoms, Roles and Maudsley score, VAS, Intensity grade, Oedema, Presence of heel spur in X-ray |
| Yin et al 2019 | Age (years), Gender, Body mass index, affected bilateral side, duration of symptoms, Roles and Maudsley score, VAS, Intensity grade, Oedema, Presence of heel spur in X-ray |

Figure: Guide for adjustments to the quality of evidence for prognosis. This diagram is adapted from Huguet et. al (2013). * In this review, moderate level of evidence is the starting point for outcome prediction research or explanatory research aimed to identify associations between potential prognostic factors and the outcome (Huguet et. al., 2013).

**References**

1. Azuero A. A note on the magnitude of hazard ratios. Cancer. 2016;122(8):1298-9.

2. Rubinstein ML, Kraft CS, Parrott JS. Determining qualitative effect size ratings using a likelihood ratio scatter matrix in diagnostic test accuracy systematic reviews. Diagnosis. 2018;5(4):205-14.

3. Menard S. Applied logistic regression analysis: Sage; 2002.
